# *Plasmodium falciparum* malaria co-infection with tick-borne relapsing fever in Dakar

**DOI:** 10.1186/s12936-017-1682-6

**Published:** 2017-01-11

**Authors:** Mamadou A. Diallo, Baidy S. Kane, Mouhamadou Ndiaye, Mouhamed Dieng, Khadim Diongue, Aida S. Badiane, Mame Cheikh Seck, Daouda Ndiaye

**Affiliations:** 1 Laboratoire de Parasitologie-Mycologie, Hôpital Aristide Le Dantec, Université Cheikh Anta Diop de Dakar, Avenue Cheikh Anta Diop, Fann, BP 5005, Dakar, Senegal; 2 Service de Médecine Interne, Hôpital Aristide Le Dantec, Université Cheikh Anta Diop de Dakar, Avenue Cheikh Anta Diop, Fann, BP 5005, Dakar, Senegal

**Keywords:** Malaria, Borrelia, Fever, Diagnostic, Co-infection

## Abstract

**Background:**

West African tick-borne relapsing fever (TBRF) due to *Borrelia crocidurae* and malaria are co-endemics in Senegal. Although expected to be high, co-infections are rarely reported. A case of falciparum malaria and *B. crocidurae* co-infection in a patient from Velingara (South of Senegal) is discussed.

**Case:**

A 28 year-old-male patient presented to Aristide Le Dantec Hospital for recurrent fever. He initially presented to a local post health of Pikine (sub-urban of Dakar) and was diagnosed for malaria on the basis of positive malaria rapid diagnostic test (RDT) specific to *Plamodium falciparum*. The patient was treated as uncomplicated falciparum malaria. Four days after admission the patient was referred to Le Dantec Hospital. He presented with fever (39 °C), soreness, headache and vomiting. The blood pressure was 120/80 mmHg. The rest of the examination was normal. A thick film from peripheral blood was performed and addressed to the parasitology laboratory of the hospital. Thick film was stained with 10% Giemsa. Trophozoite of *P. falciparum* was identified at parasite density of 47 parasites per microlitre. The presence of *Borrelia* was also observed, concluding to malaria co-infection with borreliosis.

**Conclusions:**

Signs of malaria can overlap with signs of borreliosis leading to the misdiagnosis of the latter. Thick and thin smear or QBC test or molecular method may be helpful to detect both *Plamodium* species and *Borrelia*. In addition, there is a real need to consider co-infections with other endemics pathogens when diagnosing malaria.

## Background

In Senegal, several areas are endemic for tick-borne relapsing fever (TBRF) and the causal agent is *Borrelia crocidurae*, which is transmitted by the tick, *Ornithodorus sonrai* [1, 2]. The geographic distribution of TBRF is linked to drier climates [2, 3]. However, in Senegal, important extension towards the south due to climate change was observed [1, 3], and both malaria and tick-borne relapsing fever (TBRF) due to *B. crocidurae* are endemic [4]. When there is no clinical evidence of other disease, falciparum malaria is the first suspected cause of fever in Senegal. However, borreliosis is also a major cause of fever in Senegal [5, 6]. Thus, co-infections of malaria-borreliosis are expected to be high but are rarely reported. A patient diagnosed for malaria and TBRF is discussed.

## Case presentation

A 28-year-old-male patient presented on June 9, 2016 to the health post of Pikine (sub-urban of Dakar) for headache, vomiting, diarrhea, low back pain and arthralgia. Suspecting malaria, an HRP2-based RDT specific to *Plasmodium falciparum* was performed. The test was positive for falciparum malaria. Following the Senegalese national policy guidelines for the treatment for uncomplicated malaria, the patient was given artemether 20 mg + lumefantrine 120 mg at admission and every 8 h for a total of six doses. Four days later, the patient was referred to Aristide Le Dantec Hospital. He presented with fever (39 °C), soreness, abdominal pain and vomiting.

The onset of symptoms had occurred brutally 4 days before, marked by intense and not radiating back pain without triggers or calming factors. There was also continuous soreness and joint pain without any particular pattern, associating intense asthenia, which is temporarily relieved by usual analgesics. There was also diffuse headache, without axial stiffness. There was no photophobia nor phonophobia, associating with vomiting and nausea, two to three fluids diarrhoeas that lasted 3 days without fever nor concept of eating suspected food and no similar cases in the entourage.

**Fig. 1:**
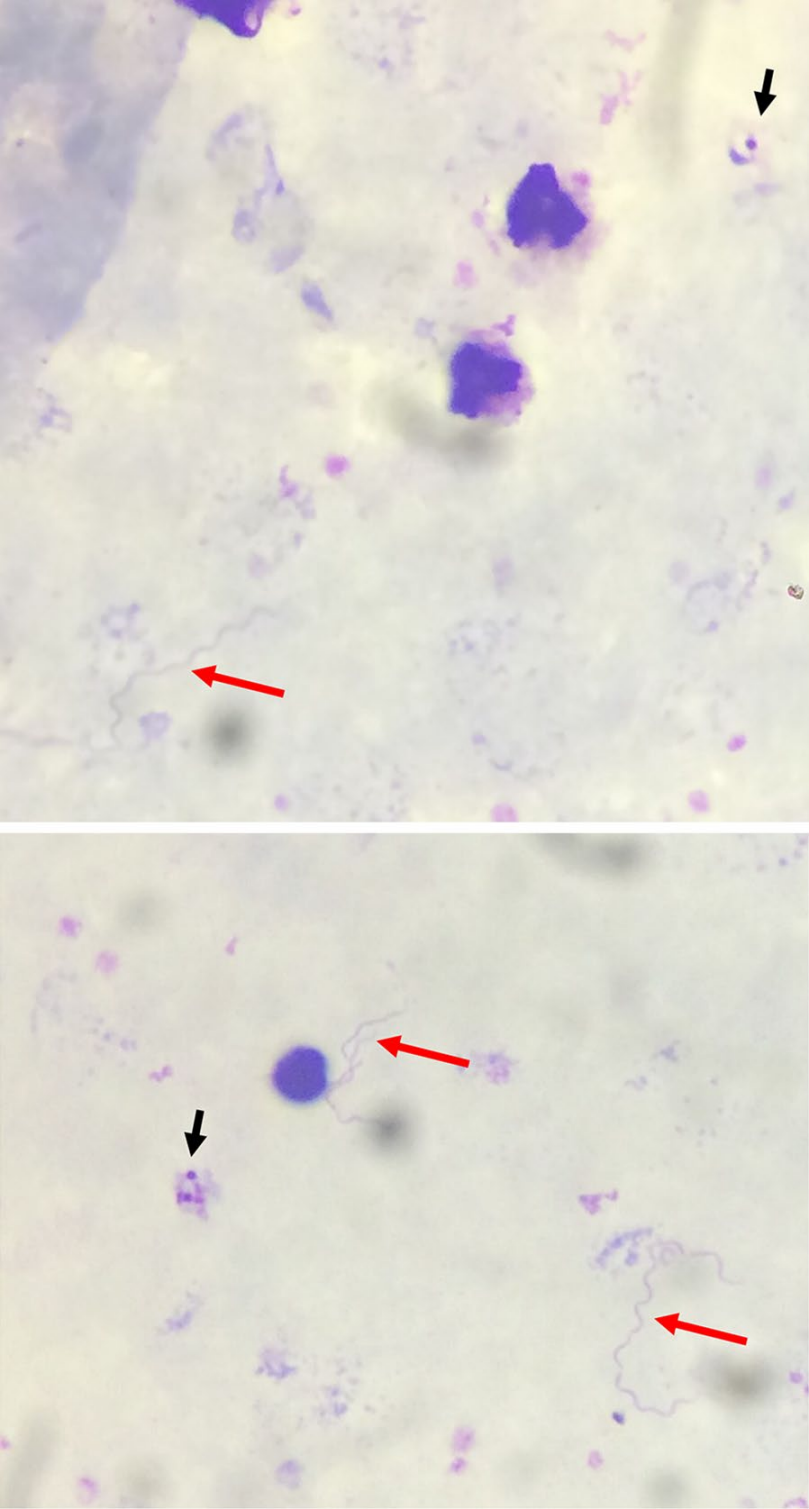
Giemsa-stained thick blood smears showing trophozoite of *P. falciparum* (*black arrow*) and spirochetes of Borrelia (*red arrow*)

Investigations shown that the patient was from Velingara (south of Senegal) and had come to Dakar 2 months earlier. He had no previous medical history. The blood pressure was 120/80 mmHg. There was no sign of clinical anaemia. The rest of the examination was normal. Laboratory test showed thrombocytopenia (platelets 40 × 10^9^/mm^3^) as the only abnormal haematological test. A thick film from peripheral blood was performed and sent to the parasitology laboratory of Le Dantec Hospital. Thick film was stained with 10% Giemsa. Trophozoite of *P. falciparum* was identified at parasite density of 47 parasites per microlitre. In addition, spirochetes of *Borrelia* were observed indicating a malaria co-infection with borreliosis (Fig. 1).

Treatment consisted of doxycycline 200 mg daily during 14 days. No further symptoms were noted. Effectiveness of treatment was confirmed by negative microscopy for both malaria parasites and spirochetes.

## Discussion

Malaria co-infection with borreliosis, rarely reported, poses diagnostic challenge [4, 7, 8].

Tropical borreliosis is often disregarded [9] and is often confused with malaria, which affects the same people and presents with similar symptoms [5]. The disease called relapsing fever was considered rare until the late 1980s, before it was demonstrated that in many rural areas of Senegal, tick-borne borreliosis was the second cause of febrile diagnosis (after malaria) [1]. With an average annual incidence of 11% for over 14 years, this is the highest rate measured in Africa for a bacterial disease [10].

Borreliosis is a major public health problem in most rural areas of Senegal. However, although it is the most common bacterial disease, it remains relatively unknown by the healthcare personnel. Most likely, misdiagnosed co-infections are believed to be malaria resistant to anti-malarial drugs [5]. Also, malaria is often diagnosed on the basis of only clinical symptoms and positive RDT, not microscopic examination of thick and thin smear, which increases misdiagnosis of possible co-infections. In addition, *B. crocidurae* is present in the blood in small quantities. It is detectable in the blood samples by a trained microscopist and only during peaks of fever [6]. In the co-infection *Borrelia*-*Plasmodium*, when examining thin or thick smears, high density Plasmodium may obscure the presence of the spirochetes, making it difficult to detect the co-infecting bacteria [4]. In this case, conversely, low parasite density of *P. falciparum* was observed probably due to parasite clearance after effective treatment just after the patient was diagnosed by malaria RDT.

Routinely, the diagnosis of both tick-borne relapsing fever and malaria is based on the detection of the pathogen in the patient’s peripheral blood. *Borrelia* and malaria parasites can be observed in Giemsa-stained thick/thin smears or fluorescence microscopy after centrifugation of blood in capillary tubes and staining with acridine orange (quantitative buffy coat [QBC] analysis) [3]. *Borrelia* can also be observed in dark field or phase contrast microscopy [3]. Positivity thresholds of thin and thick smear blood are respectively estimated at 10^5^ and 10^4^ spirochetes per milliliter of blood [4]. Giemsa method has sensitivity estimated at 25% of the reference technique (the intraperitoneal inoculation of mice is the gold standard in the diagnosis of TBRF) [6, 11]. Long and careful examination of a thick smear made at a feverish peak achieves the sensitivity from 50 to 70%.

Quantitative Buffy Coat (QBC test) is a technique for rapid detection of malaria parasites with good sensitivity and specificity. Also the QBC is very useful in *Borrelia* detection, allowing the technician to examine a larger volume of blood. With a positivity threshold of 10^3^ spirochetes/ml, this technique is 100 times more sensitive than the blood smear in the diagnosis of borreliosis [4].

Velingara is out of the distribution area of tick-borne borreliosis in Senegal [2]. Thus, the patient described here was most probably infected in Pikine (Dakar), where malaria is endemic and where *O. sonrai* is abundant in burrows of mice and rats [2]. Transmission of borreliosis is well known to occur in all regions of Senegal north of the Gambian border [2, 5], explaining the high probability for people to be co-infected by *Plasmodium* and spirochetes. Moreover, the patient stayed in Dakar for 2 months that largely exceeds the incubation period (4–14 days) for relapsing fevers [12].

Since RDTs are provided to all health clinics in Senegal, any positive test is systematically treated as malaria without considering possible co-infections. Incorrect treatment may result in persisting symptoms leading the physician to wrongly conclude drug resistant malaria. Prompt diagnosis of TBRF and appropriate treatment would avoid developing complications such as severe meningoencephalitis [3]. The recommended treatment for borreliosis is doxycycline 100 mg twice daily or erythromycin 500 mg every 6 h per os [7].

## Conclusions

This case illustrates the importance of effective and accurate diagnostic testing in tropical area where co-infections are expected to be high. TBRF should be considered in the diagnosis of every febrile patient returning from endemic areas even when malaria RDT is positive. Diagnosis relies upon routinely microscopic examination of thick and thin smear. QBC might increase the sensitivity and when available, molecular tools are highly effective in detecting spirochetes.
